# Three-Year Monitoring of Microorganisms’ Composition and Concentration in Atmospheric Aerosols of Novosibirsk City and Suburbs

**DOI:** 10.3390/microorganisms12102068

**Published:** 2024-10-15

**Authors:** Irina Andreeva, Aleksandr Safatov, Olga Totmenina, Sergei Olkin, Maxim Rebus, Galina Buryak, Tatiana Alikina, Olga Baturina, Marsel Kabilov

**Affiliations:** 1 Department of Biophysics and Ecological Researches, Federal Budgetary Research Institution—State Research Center of Virology and Biotechnology VECTOR, Federal Service for Surveillance on Consumer Rights Protection and Human Welfare, 630559 Koltsovo, Novosibirsk Region, Russia; andreeva_is@vector.nsc.ru (I.A.); totmenina_od@vector.nsc.ru (O.T.); olkin@vector.nsc.ru (S.O.); rebus_me@vector.nsc.ru (M.R.); buryak@vector.nsc.ru (G.B.); 2 Genomics Core Facility, Institute of Chemical Biology and Fundamental Medicine, Siberian Branch of the Russian Academy of Sciences, 630090 Novosibirsk, Russia; alikina@niboch.nsc.ru (T.A.); baturina@niboch.nsc.ru (O.B.); kabilov@niboch.nsc.ru (M.K.)

**Keywords:** Novosibirsk, atmospheric aerosol, microorganisms, biodiversity, concentration, pathogenicity, metabarcoding, 16S rRNA

## Abstract

The atmospheric environment is formed under the influence of local and distant sources as a result of horizontal and vertical transport. In the present work, microbiological analysis of 604 samples of atmospheric aerosol collected in the period from September 2020 to September 2023 at four sites differing in anthropogenic load, located in Novosibirsk and the region, was carried out. Day and night aerosol samples were collected during 12 h every two weeks by filtration using Sartorius reinforced Teflon membranes, then sown on a set of nutrient media. The taxonomic affiliation of the isolated microbial isolates was determined based on phenotypic characteristics and analysis of 16S rRNA gene nucleotide sequences. Changes in the composition and concentration of culturable microorganisms depending on the season, time of day, and site of aerosol sampling were observed. In winter, lower fungi and bacteria of the genera *Bacillus*, *Staphylococcus*, *Micrococcus* dominated with an average concentration from zero to 12.5 CFU/m^3^ of aerosol. In the warm period, the concentration and diversity of cocci, spore-forming and non-spore-forming bacteria, actinomycetes, and fungi (up to 1970 CFU/m^3^), among which pathogenic microorganisms were found, increased sharply in aerosols. The use of 16S metabarcoding techniques has greatly expanded the range of aerosols’ microbial diversity detectable.

## 1. Introduction

The atmospheric environment of cities is formed under the influence of powerful sources of various origins, local and remote, as a result of horizontal and vertical air mass transport. Environmental pollution in large industrial cities has become one of the pressing environmental problems of our time. A high level of anthropogenic impact not only determines the urban atmosphere, but influences the atmosphere of the suburbs and the region also. According to statistical data, in the balance of emissions of pollutants into the atmosphere of the Novosibirsk region, the city of Novosibirsk is responsible for 30.2% of the volume of emissions [1]. The level of atmospheric pollution in Novosibirsk is estimated based on data from 10 stationary observation posts and is characterized as high, which is primarily due to the increased content of suspended matter and a number of toxic compounds in the air [1]. A significant part of atmospheric aerosols consists of bioaerosols containing bacteria and archaea, spores and fragments of fungi, pollen, viruses, algae and cyanobacteria, biological crusts and lichens, and others, such as fragments of plants or animals and detritus, which are in a free or sorbed state. In recent decades, interest in biological aerosols has increased significantly [2]. It has been shown that biological particles can have a significant impact on clouds and precipitation and thus influence the hydrological cycle and climate [3,4]. It has been found that biological particles are associated with a variety of negative health effects, ranging from infectious diseases to acute toxic effects, allergies, asthma, and even cancer [5].

It should be noted that there is a widespread presence of microorganisms that are multiresistant to antibiotics, which cause some human infections. These microorganisms may contaminate the atmosphere and are dangerous for humans [6,7]. The literature presents numerous works devoted to various aspects of the habitat of microorganisms in the air; as a rule, studies were conducted for the atmosphere of settlements and large cities in the middle latitudes and southern zones [8,9,10,11,12,13,14,15,16,17]. The northern territories remain the least studied; few works are known using molecular biological methods [18], and few complex studies of atmospheric aerosols in the south of Western Siberia and Arctic territories are known [19,20,21,22]. Generally, southern latitudes are characterized by the predominance of the fungi in aerosols; in middle latitudes bacteria often predominate. But due to efficient aerosol transportation between different regions, observed concentrations of microorganisms in atmospheric aerosols in various regions differ not too much. The atmospheric microbiota of cities in the Siberian region, including Novosibirsk and the Novosibirsk region, are poorly studied. Since most of the atmospheric microbiome still cannot be cultivated in laboratory conditions, studying the diversity of microorganisms using high-throughput sequencing methods, which allows the identification of unculturable microorganisms in the air environment, is of utmost importance [23,24].

Microorganisms enter the atmosphere in the form of aerosol particles from almost all available surfaces, including soil, water, and vegetation [25]. For the survival of bacteria and fungi in unfavorable atmospheric conditions, the ability to form cellular structures that are resistant to such environmental factors as low humidity, high salt content, UV irradiation, and low environmental temperatures is of great importance. Microorganisms that have such protection primarily include cocci of the genera *Micrococcus*, *Staphylococcus*, bacteria of a number of taxa that form spores, and micromycete fungi. All over the world, microorganisms found in the internal air environment of transport, hospitals, residential and work premises are being actively studied, which, having a specific diversity of microbiome with air flows, can contaminate the atmosphere. The facts of their negative impact on human health (microorganisms in aerosols can cause or provoke infection diseases, allergic reactions, toxic effects, etc.) have been confirmed by numerous works [26,27,28]. In connection with the above, studying the biodiversity of the microbiota of the atmosphere of the urban environment of the northern regions is important.

The main purpose of this work is to study the composition and concentration of microorganisms in atmospheric aerosols of Novosibirsk city and the suburbs during three years of monitoring depending on the season, time, and place of taking the aerosol sample. It was also planned to assess how different methods of obtaining isolates affect their representativeness in relation to 16S metabarcoding results.

Microorganisms’ culturing can be useful for several reasons. Firstly, in the case of air we are dealing with tiny amounts of material, especially in winter, which makes the use of metagenomic sequencing very problematic. Secondly, in the case of cultivation, it is possible to test the isolate for its beneficial properties immediately. Thirdly, using cultivation we have already discovered several new species of bacteria (the material will be included in the next article).

## 2. Materials and Methods

**Figure 1:**
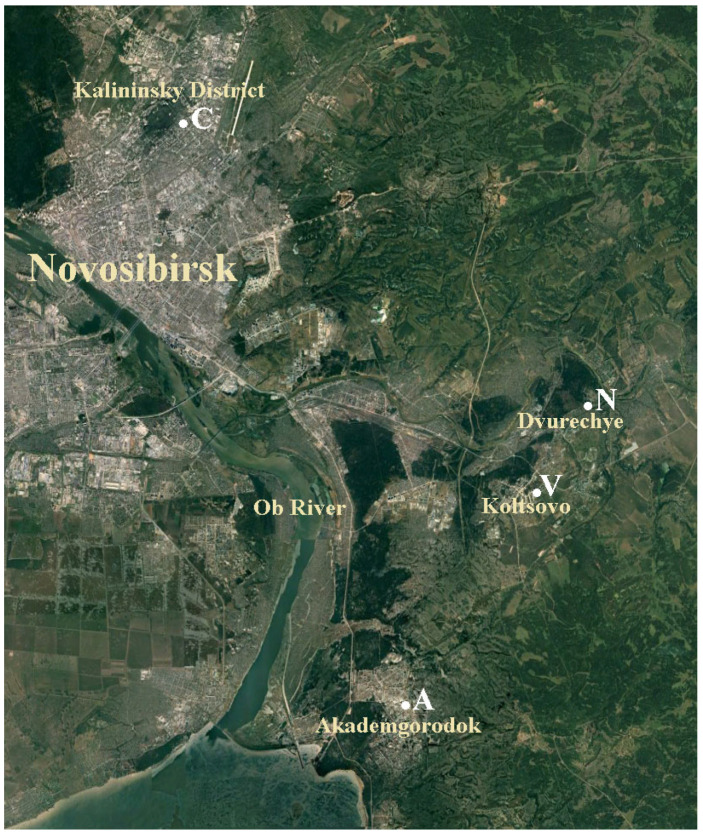
Sampling sites of atmospheric aerosols in Novosibirsk and the region. Explanations are in the text.

Atmospheric aerosol samples were obtained by filtration method using Sartorius reinforced Teflon membranes (membrane # FIL 1546 with diameter 47 mm and pore size 1.2 μm) and Hopar compressors with flowrate 1.75 m^3^ per hour. Aerosol sampling sites: A—Akademgorodok (Sovetsky district of Novosibirsk); C—Novosibirsk (Kalininsky district); N—settlement Dvurechye, Novosibirsk suburbs; V—settlement Koltsovo, Novosibirsk suburbs (Figure 1). Climatic conditions during sampling are presented in Table S1. For desorption of microorganisms, the filters were placed in tubes with 5 mL of sterile physiological solution (0.9% NaCl) and shaken for 30 min at room temperature on an orbital shaker (OLab SIP-05P2, Moscow, Russia), then a vortex (Vortex-Genie, Zurich, Switzerland) was applied for 2 min. The obtained suspension was used for microbiological studies. The resulting suspensions were sown on a set of complete and mineral nutrient media differing in composition and pH value as described previously [29]: LB medium (Difco, Franklin Lakes, NJ, USA); depleted LB medium (1:10 dilution); soil agar (SA) [30], thioglycol medium(Tg); starch-ammonia agar (SAA); and Sabouraud medium (Sm, produced by the Federal Budgetary Institution of Scientific Research Center of PMB, Obolensk, Russia). The samples were seeded onto the surface of Petri dishes with different culture media and then were incubated for 24 h at a temperature of 28–30 °C and then at room temperature for seven days. Three parallels of initial sample dilutions were used (usually 0 to 3 dilutions of 1:10) to determine CFUs per ml. The identified isolated colonies of microorganisms were used to determine the composition and concentration of aerosols of bacteria and fungi in the studied samples, indicated by the Km index. A comparative quantitative assessment of the concentration of microorganisms in the sample is given as CFU/m^3^.

In addition, the method of direct natural microorganisms’ deposition from atmospheric air onto the surface of nutrient media was used. To do this, dishes with a set of media (CAA, SA, LB, and LB 1:10 agar media, Sabouraud medium) with the lids removed were exposed in the open air, under conditions similar to those used on 9 August 2021 during standard sampling (Koltsovo, samples V1 and V2 from 9 July 2021). Three temporary exposures of cups with media were used—6 h in the daytime (9:00–15:00, 9 August 2021); day—24 h (9:00 9 August 2021–9:00 9 September 2021); and 12 h at night (19:00 9 August 2021–07:00 9 September 2021). The dishes seeded in this way were kept for 24 h at a temperature of 28–30 °C and then at room temperature for seven days. The temperature of 28 °C is a borderline temperature: at this and higher temperatures pathogenic microorganisms grow actively; for saprotrophic microorganisms, this temperature and lower temperatures are also acceptable. Additional incubation of sieves at room temperature for more than 10 days allows the detection of microorganisms that grow slowly and are suppressed by higher temperatures. This approach to cultivation leads to a more complete extraction of representatives of the aerosol microbiome. The use of nutrient media of different composition makes this possibility more effective. Total washes with a neutral buffer of all grown colonies from two plates with SA (samples w1 and w3) and one plate with LB agar (1:10) (sample w2) were also prepared for genomic research.

The morphological properties of isolated microorganisms were studied using light microscopy of fixed stained cells and intravital preparations observed by phase contrast using an Axioskop-40 microscope (Karl Zeiss, Gottingen, Germany). Physiological and biochemical characteristics were studied using standard methods [31,32].

The methods of measurement of bacteria enzymatic activities were described in detail in [33].

The taxonomic positions of bacteria isolated from aerosol samples (Km, Sb) were determined by analysis of nucleotide sequences of the 16S rRNA gene fragments. DNA from the bacteria studied was isolated using the GeneJET Genomic DNA Purification Kit (Thermo Fisher). Next, using primers 27F (AGAGTTTGATCMTGGCTCAG) and 1492R (GGTTACCTTGTTACGACTT), an amplicon corresponding to the 16S rRNA gene was obtained. The PCR used Phusion Hot Start II Polymerase (NEB) with the program: 98 °C-1’, [98 °C-10”, 62 °C-15”, 72 °C-45”] × 33 cycles, 72 °C-7’. Determination of nucleotide sequences was carried out on an ABI Prism 3130XL automatic sequencer (Applied Biosystems, Carlsbad, CA, USA) using a set of BigDye^®^ Terminator v3.1. Cycle Sequencing Kit (Applied Biosystems, USA) reagents and primers 27F and 1492R. Forward and reverse reads were stitched to create a contig in CLC Main Workbench (Qiagen). Contig analysis to determine the taxonomy of the closest homologues was performed using nucleotide blast (https://blast.ncbi.nlm.nih.gov/Blast.cgi accessed on 24 July 2024) using the Nucleotide Collection (nt) database.

The DNeasy PowerSoil Kit (Qiagen) was used for total DNA extraction from membrane (205, 206 samples) or all grown colonies from plates (w1–w3) according to the manufacturer’s instructions, the bead-beating performed with the TissueLyser II (Qiagen) for 10 min at 30 Hz. The V3-V4 region of the 16S rRNA genes was amplified with the primer pair 343F (5′-CTCCTACGGRRSGCAGCAG-3′) and 806R (5′-GGACTACNVGGGTWTCTAAT-3′) combined with Illumina adapter sequences [34]. PCR amplification was performed as described earlier [35]. A total of 200 ng PCR product from each sample (mix of three technical replicates) was pooled together and purified through the MinElute Gel Extraction Kit (Qiagen, Hilden, Germany). The obtained 16S libraries were sequenced with 2 × 300 bp paired-ends reagents on MiSeq (Illumina, Sand Diego, CA, USA) in the SB RAS Genomics Core Facility (ICBFM SB RAS, Novosibirsk, Russia).

Raw sequences were analyzed with the UPARSE pipeline [36] using Usearch v.11.0.667. The UPARSE pipeline included merging of paired reads, read quality filtering (-fastq_maxee_rate 0.005), length trimming (remove less than 350 nt), merging of identical reads (dereplication), discarding singleton reads, removing chimeras, and operational taxonomic unit (OTU) clustering using the UPARSE-OTU algorithm. The OTU sequences were assigned a taxonomy using the SINTAX [37] and 16S RDP training set v.19 [38] as a reference. Taxonomic structure was estimated by the ratio of the number of taxon-specific sequence reads to the total number of sequences reads, i.e., by the relative abundance of taxa, expressed as a percentage. The Chao1 index was calculated in Usearch. Principal component analysis (PCA) was performed on the data using Python’s scikit-learn package [39].

## 3. Results

During the 3-year observation period from September 2020 to September 2023, 604 samples of atmospheric air were obtained using filtration to study the biodiversity and concentration of culturable microorganisms in atmospheric aerosols in the Novosibirsk region. When the resulting aerosol samples were sown on nutrient media, 5512 isolates’ clear cultures (these clear cultures were considered “isolates” as soon as not all of them were characterized to “strain” level) of bacteria and fungi were isolated into cultures. A collection database of microbial aeroisolates (7572 storage units) has been formed, reflecting the biodiversity of the microbiome of the atmosphere of Novosibirsk City, its suburbs, and the region, stored by low-temperature freezing as part of the collection of natural isolates of the Department of Biophysics and Ecological Research of the Federal Budgetary Institution State Research Center of Virology and Biotechnology “Vector” of Rospotrebnadzor.

The concentration of cultivated microorganisms in individual samples of the studied aerosols varied significantly, ranging from zero and several units up to 4000 CFU/m^3^, which is evidence of significant contrasting changes occurring in the atmosphere, affecting the composition of bioaerosols. The seasonal decrease in the number of detected microorganisms in the winter of northern territories covered with snow for a long time, reaching zero, can be considered natural, which does not exclude individual outbreaks of their concentration caused by transfer of air masses from distant sources, or local ones. During the cold period of the year, in aerosol samples, both in Novosibirsk and in the suburbs, representatives of the genera *Bacillus*, *Staphylococcus*, *Micrococcus,* and micromycete fungi, which belong to the group of microorganisms resistant to low humidity, high salt concentrations, temperature changes, and ultraviolet radiation, and other unfavorable environmental factors were most often found.

**Figure 2:**
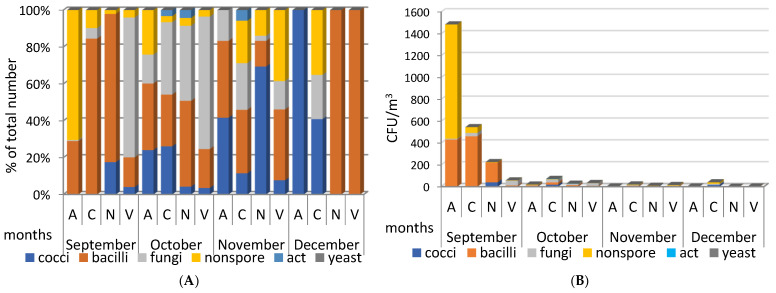
(**A**)—quantitative ratio of different groups of microorganisms from the total number of isolated microorganisms (%); (**B**)—concentration (CFU/m^3^) of culturable microorganisms of different groups in samples of atmospheric aerosols of Novosibirsk and the region in 2020. “nonspore”—non-spore-bearing bacteria; “act”—actinomyces. Aerosol sampling sites: A—Akademgorodok (Sovetsky district of Novosibirsk); C—Novosibirsk (Kalininsky district); N—settlement Dvurechye, Novosibirsk suburbs; V—settlement Koltsovo, Novosibirsk suburbs (Figure 1).

We began studies of the microbiome of atmospheric aerosols collected at stationary sites A, C, N, and V in September 2020. In terms of the number and composition of isolated microorganisms, the studied September aerosols differed markedly from aerosols of later periods (Figure 2).

In September 2020, the highest concentration of microorganisms (up to 1500 CFU/m^3^), represented mainly by non-spore-bearing and spore-forming bacteria, was found in aerosol samples taken at site A. In aerosols from sampling sites C and N, spore-forming bacteria predominated, but with a significantly lower total number of isolated microorganisms; site V was characterized by a reduced concentration, with a predominance of fungi (Figure 2). In subsequent months until the end of 2020, in the studied aerosols, both daytime and nighttime, a low concentration of isolated microorganisms remained (from zero to few tens of CFU/m^3^), with a small peak in the nighttime aerosol sampled at site C in October.

**Figure 3:**
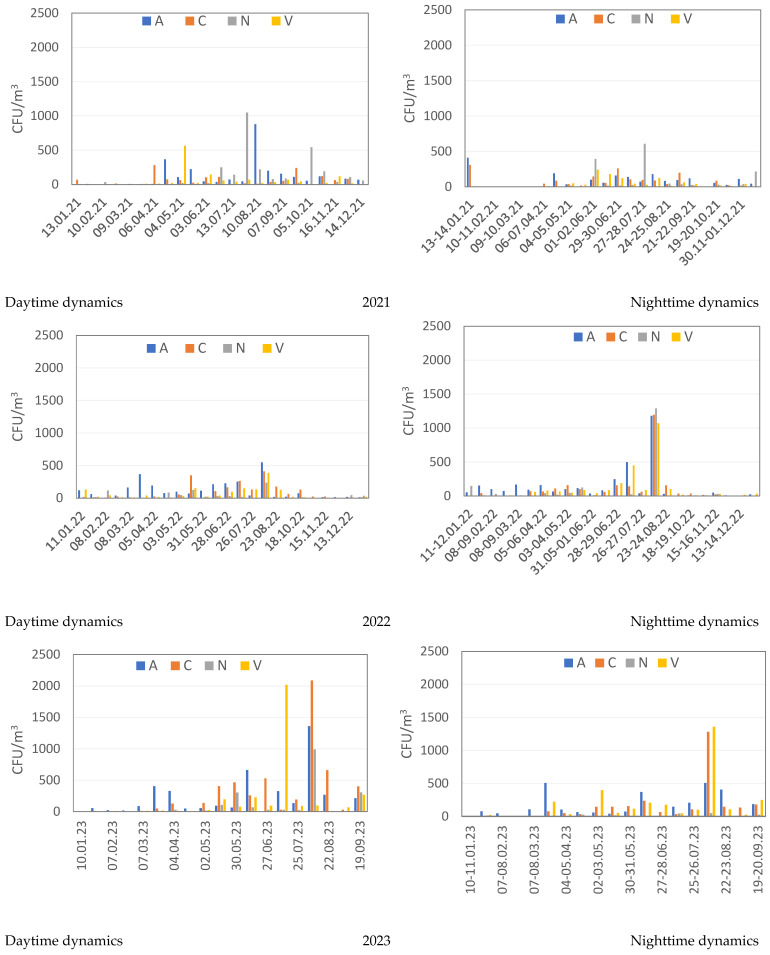
Daily dynamics of the concentration of culturable microorganisms in the studied atmospheric aerosols (CFU/m^3^). Aerosol sampling sites: A—Akademgorodok (Sovetsky district of Novosibirsk); C—Novosibirsk (Kalininsky district); N—settlement Dvurechye, Novosibirsk suburbs; V —settlement Koltsovo, Novosibirsk suburbs (Figure 1).

Figure 3 shows seasonal and daily changes in the concentration of microorganisms in the studied atmospheric aerosols during 2021–2023, determined for four sampling sites A, C, N, V during the day and night. Nighttime concentrations of identified culturable microorganisms are generally slightly lower than daytime concentrations, but isolated outbreaks of numbers were possible that significantly exceeded their average values, as, for example, in August 2022 and 2023. The highest concentration of microorganisms is observed for all sampling sites from June to September, which may be due to the increased content of suspended particles contaminated with microorganisms at this time. In many aerosol samples, the culturable fungi were dominated by widespread micromycetes of the genera *Penicillium*, *Aureobasidium*, *Aspergillus*, *Alternaria*, *Cladosporium*, and a number of others. The highest concentration of fungi, reaching up to 1550 CFU/m^3^ in individual aerosol samples, was observed in spring–summer and early autumn samples. During the winter period, a minimum concentration is observed in aerosol samples (Figure 3).

In aerosol samples from 2022 and 2023 during the warm period, the lowest concentrations of culturable microorganisms were found in samples taken in the village. On the contrary, the number of detected culturable microorganisms at site N was the highest in 2021, in comparison with the concentrations found at other sampling sites. The highest total number of culturable fungi, spore-forming and non-spore-forming bacteria was revealed reaching 2100 CFU/m^3^ of aerosol suspension during the summer period of 2023, in samples from sampling sites C and V (Figure 3), significantly exceeding similar values in 2021 and 2022.

**Figure 4:**
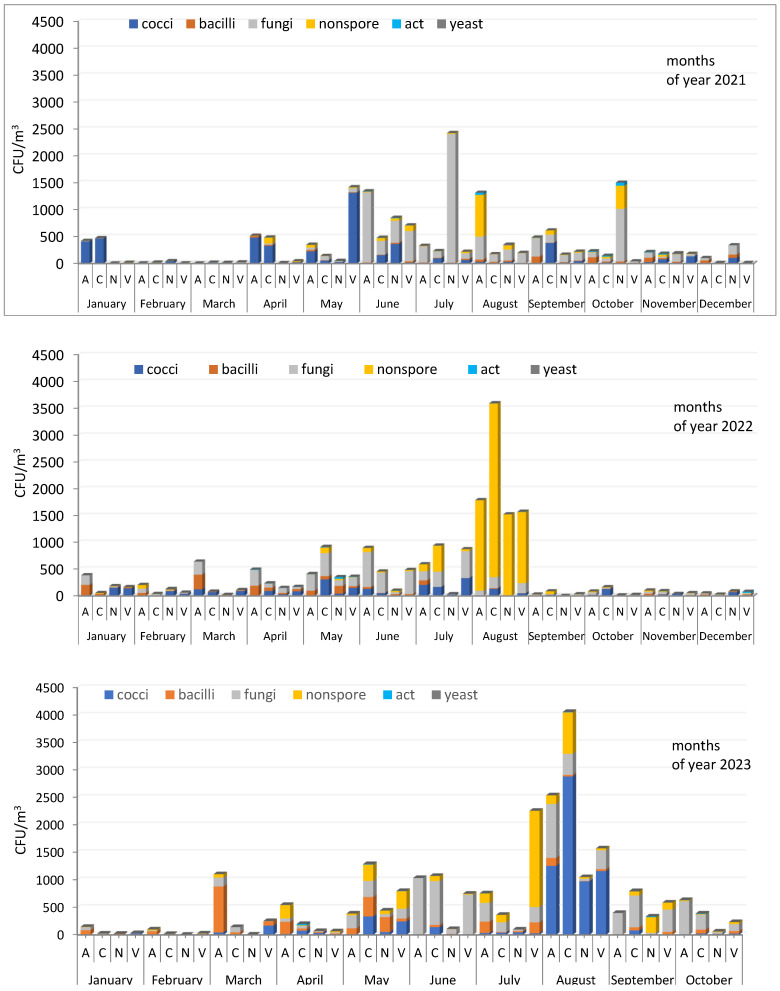
Concentration and composition of culturable microorganisms isolated from atmospheric aerosol samples from Novosibirsk and the region in 2021–2023. Aerosol sampling site designations are the same as in Figure 3.

The ratios of microorganisms of different groups isolated in 2021, 2022, and 2023, expressed as % of the total number detected, are shown in Figure S1. All three years differ from each other in the composition of the dominant culturable microorganisms in aerosols, while the seasonal dependence of its total concentration is similar—reduced in the cold season and increased in the warm period. Certain precise patterns in the ratio of individual taxonomic groups in the studied aerosol samples were not identified. However, some similarities in the composition of microorganisms of a number of samples are observed: January 2021—cocci predominated at sites A and C; January 2022 and 2023—bacilli and fungi were found in similar proportions; August 2022—non-spore-forming bacteria dominated at all sampling sites; in June of all three years, with a few exceptions, fungi were the predominant species in the aerosol samples; the same thing was observed in September for sampling sites A and N; for site C, a similar situation was found only for September 2023 (Figure 4 and Figure S1).

The results of phenotypic and genetic characteristics studies made it possible to identify, among culturable isolates found in samples of atmospheric aerosols, non-spore-forming bacteria of the genera *Agrococcus*, *Nocardiopsis*, *Planomrobium*, *Planococcus*, *Pseudomonas*, *Arthrobacter*, *Brevundimonas*, *Curtobacterium*, *Psychrobacter*, *Stutzerimonas*, spore-forming bacteria of the genera *Bacillus*, *Paenibacillus*, *Priestia*, *Lysinibacillus*, *Peribacillus*, *Metabacillus*, *Solibacillus*, etc., as well as bacteria of the genera *Kocuria*, *Planococcus*, *Mammaliicoccus*, *Macrococcus*, *Staphylococcus*, *Sporosarcina,* and a number of others. The strains were tested for the presence of secretion of a number of enzymes, partly for antibiotic activity also. The results of identification of 270 culturable bacteria to species level based on the analysis of phenotypic characters and 16S rRNA gene sequences (97.5–100% similarity to GenBank data) are shown in Table S2.

There are both saprotrophic bacteria and bacteria that can cause diseases, such as *Kocuria rosea*, *Staphylococus aureus*, *Bacillus cereus*, *Staphylococus pasteuri*, *Staphylococus saprophyticus*, *Staphylococus equorum*, *Acinetobacter lwoffii*, and a number of others among the isolates found. The presence of aggressive enzymes or the ability to secrete enzymes such as hemolysins, alkaline phosphatase, lecithinase, plasma coagulase, and gelatinase which contribute to the development of the infectious process was revealed for some isolates. The demonstrated potential pathogenic threat, characteristic of many isolated representatives of these genera [26,27], suggests the need to control them in the atmospheric air in order to avoid the occurrence of infectious diseases in the population.

It is known that the results of studying the same samples of air aerosols using metagenomic methods and standard cultivation methods differ significantly in the general composition and identification of the dominant groups of microorganisms [32,40,41,42], which is also further confirmed by the results obtained in this work. However, it was interesting to see how much the groups of microorganisms identified by different methods overlap with each other.

When seeding aerosols onto agar media, it is possible to form a large number (up to 1000 or more) of small and minute colonies, which are difficult to accurately count by microscopy, and sift out for further research. Most often, such primary colonies, which are not further cultured, are formed when aerosols are sown on mineral media, media with a small amount of organic matter, while they are absent on complete media. In particular, we observed this kind of results when sowing aerosols from the autumn period from 7–8 September 21 (sampling site in the settlement Koltsovo) using the method of direct deposition on soil agar (samples V1-1, V2-1, Table 1).

The total number of microorganism colonies isolated by this method significantly exceeds the number isolated by the standard filtration method adopted in this study (Table 2). A similar difference is observed in the diversity allocated. It is possible that the filtration method used is a factor that selects the microorganisms that are most resistant to unfavorable environmental conditions, primarily fungi, gram-positive cocci, and spore-forming bacteria that are resistant, for example, to desiccation.

To compare the taxonomy of microorganisms obtained by different methods, 16S metabarcoding was carried out, as well as the identification of individual isolates using complete 16S rRNA gene sequencing. Metabarcoding was used to analyze two samples (205 and 206), representing total DNA isolated after filtering daytime and nighttime air, respectively. This method also analyzed the smallest colonies on agar media, which were prepared as total washes with a neutral buffer of all grown colonies from two plates with PA (w1 and w3) and one plate with LB agar (1:10) (w2).

Culturable bacterial isolates, designated as Sb, were isolated from individual colonies on different media using the direct sorption method, i.e., deposition of aerosol particles onto dishes with agar media for a specified time. Isolates designated Km are culturable bacteria isolated from suspensions obtained by washing filters in sterile saline (details in Section 2).

**Figure 5:**
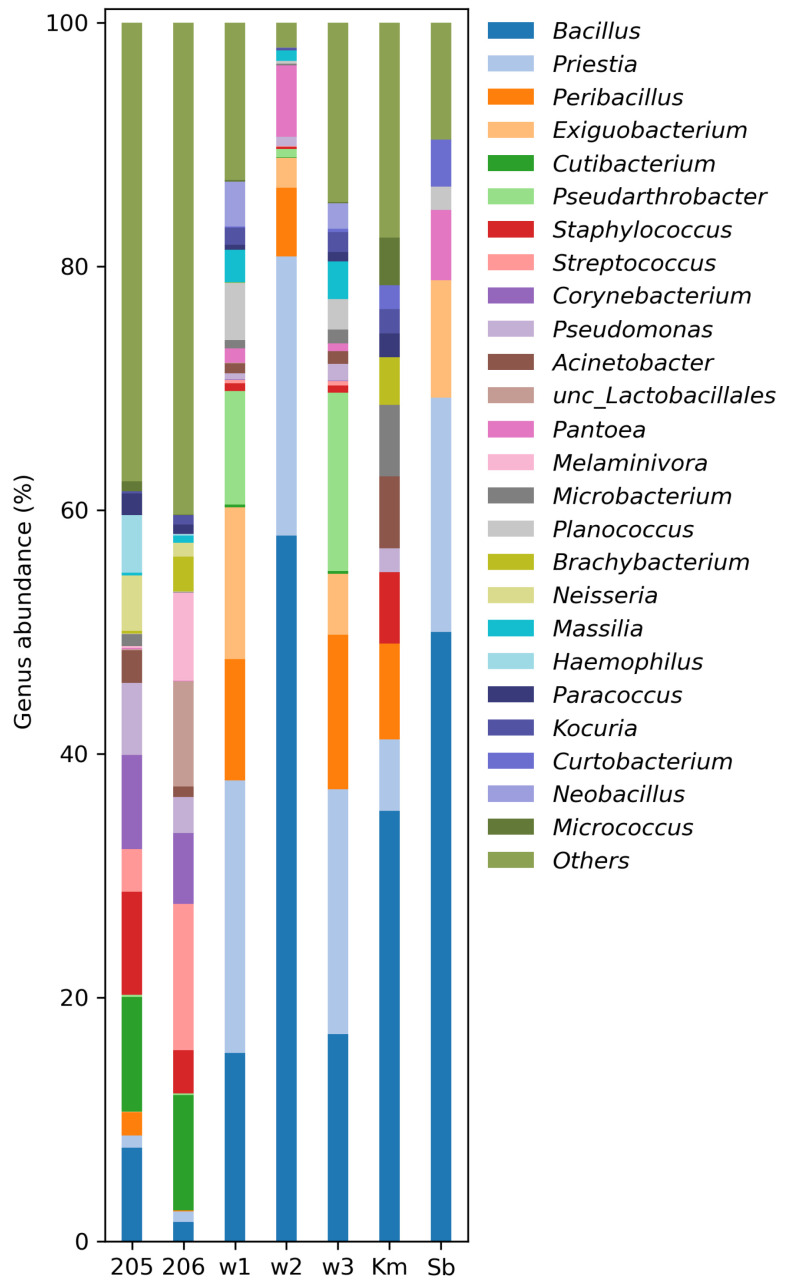
Relative abundance (%) of genera-specific 16S rRNA gene amplicon sequences in the aerosol samples. For samples 205, 206, w1–w3 metabarcoding was used, but Km and Sb were analyzed by Sanger sequencing.

The identified biodiversity of bacteria of the Sb and Km groups at the genus level, as well as in washings of the smallest colonies, is shown in Figure 5, and the distribution for other taxonomic levels is in Table S3. The taxonomic groups that are most represented in the sample of the aerosol under study are highlighted in color in Table S3.

To compare the estimated number of species in each sample, the Chao1 index was calculated using singleton and doubleton values. For a number of samples 206, 205, w1, w3, w2, Km, Sb, the following values were obtained: 379, 358, 230, 164, 123, 123, and 57, respectively. The maximum number of species, as expected, was obtained for samples 205 and 206. In the case of samples representing colony washes (w1–w3), as well as Km (from suspensions), the number of species is reduced by 1.5–3 times. The minimum index value is observed for Sb during direct deposition.

**Figure 6:**
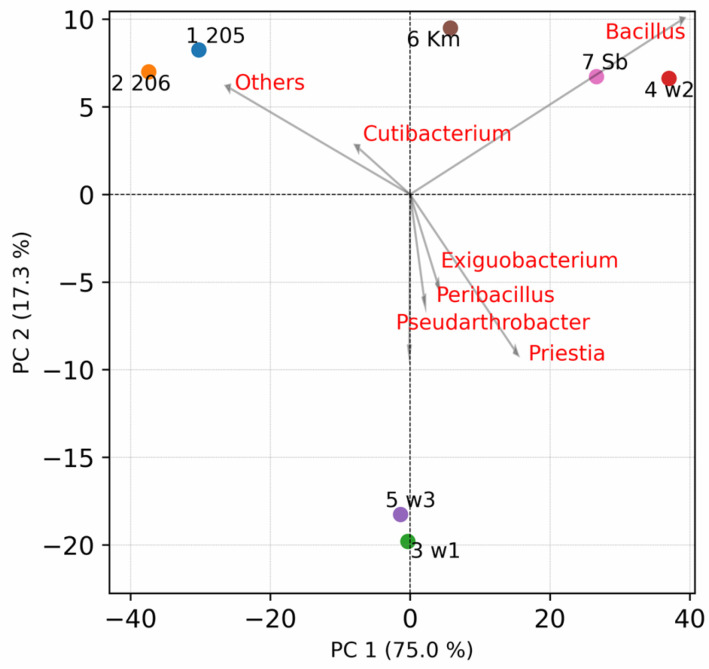
The principal component analysis of samples at the genus level. Principal component 1 and 2 explained 75.0% and 17.3% of the total variations, respectively. Red names of top seven taxa by the longest loading vector length are indicated.

The degree of similarity between the samples was assessed using principal component analysis at the genus level (Figure 6). Samples 205 and 206, in which the maximum number of genera were identified, form the first cluster. The closest distance from it is the cluster formed by the Sb and w2 samples, which are distinguished from the rest by their increased p content. Between the first two clusters there is sample Km, which turns out to be closest to samples 205 and 206 from all sites. The last sites w1 and w3 differ significantly from all others in the second component, due to the increased content of the genera *Exiguobacterium*, *Peribacillus*, *Pseudarthrobacter*, and *Priestia*. Thus, the composition of the culture medium has a significant impact on the diversity of microbial isolates detected.

**Figure 7:**
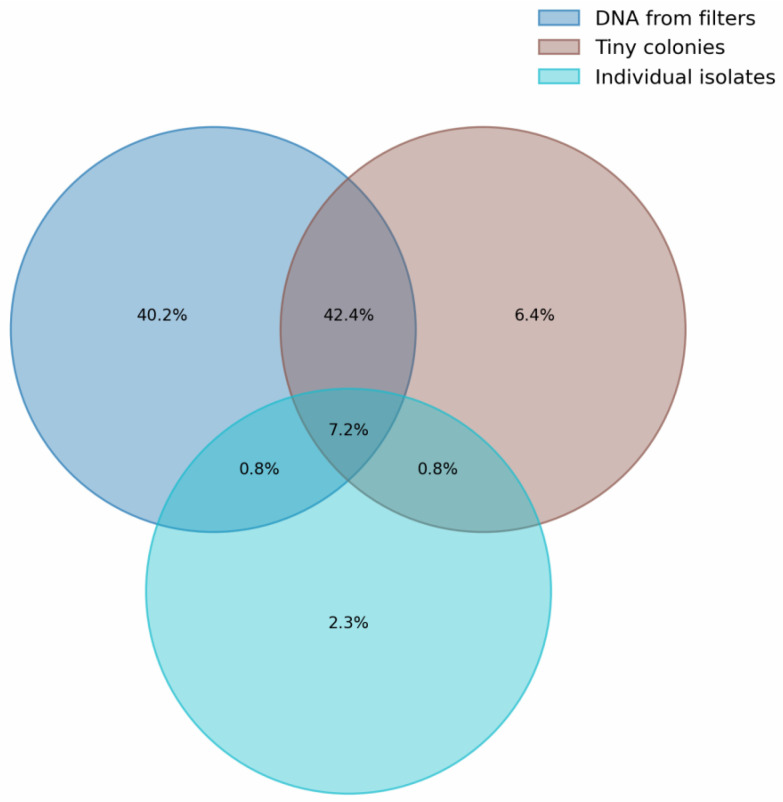
Venn diagram of genera intersection for samples analyzed by 16S metabarcoding (DNA from filters (205, 206) and tiny colonies (w1, w2, w3)) and full 16S Sanger sequencing of isolates (Km and Sb).

Figure 7 is a Venn diagram showing the intersection by genus for identified microorganisms in different sample types (Figure 6). It can be seen that about 40% of the diversity identified for samples 205 and 206 is not found in other groups. The maximum overlap (>50%) is observed between groups of samples analyzed using 16S metabarcoding, namely between DNA from filters and tiny colonies. The number of genera occurring in all groups was about 7%. It is worth noting that for a group of individual colonies, genera that do not overlap with other groups constitute only 2.3% of the total, which is 20% relative to all genera in this group.

## 4. Discussion

Seasonal changes in the composition and concentration of the microbiome of atmospheric aerosols have been observed by many authors [43,44,45]. In winter, as a rule, the lowest average concentrations of microorganisms were detected in aerosols, and the highest in summer [46]. A similar situation was found for the aerosols studied in this work. The higher summer concentrations observed in many aerosols may be due to seasonal differences in temperature and its effect on source strength and atmospheric convection [47]. Urban areas with heavy vehicle traffic have increased concentrations of aerosol particles containing microorganisms compared to quieter areas such as parks [48]. Sampling site C and, to a somewhat lesser extent, sampling site A have similar characteristics and more often than others in 2022 and 2023 had an increased concentration of microorganisms in the aerosol, at the same time, while site N, located far from industrial areas, generally had a low concentration of detectable microorganisms.

Meteorological variables such as precipitation and wind speed can also have a significant impact on surface emissions and atmospheric concentrations of microorganisms [49]. Phenomena such as heavy snowfalls or prolonged rains are a kind of cleansing shower for the atmosphere, which leads to zero values in the subsequent study of the microbiome. The effective washout of aerosol microbial cells during heavy snowfall can be significant; the concentration of detected microorganisms in the atmosphere can be very low, and in the northern regions lasts up to several subsequent weeks [50]. We observed similar phenomena when sowing aerosol samples after heavy rain or snowfall (samples from 17 January 2022, sampling sites C and V; samples from 14 February 2022, sampling site V; samples from 26 February 2022, sampling sites C and N; samples from 26 February 2022, sampling sites C and N; samples from 26 February 2021 and 9 March 2021, sampling sites A, N, C, V; samples from 18 May 2021, sampling sites A and N; sample dated 21 March 2023, sampling site N; sample dated 12 June 2023, sampling site N; and a number of others).

It should be noted that there are no patterns in the ratio of individual groups of microorganisms in the studied aerosols. In 2021, cocci were isolated more often, in 2023 bacilli; in different samples, in many cases, micromycetes, along with others, made up a significant part of the aerosol microorganisms, regardless of the site of sampling. Peak maximum concentrations in different years are represented by different microorganisms. In 2021, high numbers were more often provided by fungi, especially at the beginning and end of the growing season of plants with which they are closely related, in 2022 by non-spore-forming bacteria, in 2023 by spore-forming bacteria, which indicates a high variability and mobility of the atmospheric microbiome composition.

The similarity in the composition of microorganisms in individual samples seems more random than natural. The year 2023 was characterized by long dry periods, which may have contributed to higher levels of microorganisms in aerosols compared to previous years due to higher concentrations of suspended dust particles in the air. In the first 9 months of 2023, the second most increased level of air pollution with suspended substances was most often recorded in Novosibirsk. At the same time, the third-highest level in the city was observed for three months in a row from June to August. This is evidenced by data from the West Siberian meteorological service, which can also to some extent explain the increased concentration of microorganisms in aerosols in 2023 compared to 2020–2022. Transport from distant sources may also contribute to the observed episodic increases in concentrations [51,52,53].

Fungi of the genera *Penicillium*, *Aureobasidium*, *Aspergillus*, *Alternaria*, *Cladosporium* and bacteria of the genera *Kocuria rosea*, *Staphylococus aureus*, *Bacillus cereus*, *Staphylococus pasteuri*, *Staphylococus saprophyticus*, *Staphylococus equorum*, *Acinetobacter lwoffii*, found in aerosol samples, which can cause allergic and infectious diseases, are an indicator calling for regular microbiological monitoring of the air environment for the prevention and protection of public health [54,55]. The literature contains the results of a study of air microorganisms, indicating that exposure to a variety of microorganisms can be beneficial; for example, the microbiome of open air and living environment in childhood provides protection against asthma and immune diseases [56]. This is because the niche occupied by different taxa in the respiratory tract limits the surface area on which pathogenic bacteria thought to cause respiratory tract disease can settle [57].

Among the identified culturable microorganisms, a large number of species of spore-forming bacteria of the genera *Bacillus*, *Paenibacillus*, *Priestia*, *Lysinibacillus*, *Peribacillus*, *Metabacillus*, and *Solibacillus* were found, representatives of which are known as producers of various biologically active substances [58,59,60,61]. This work obtained primary information on enzymatic activity for the isolated cultures of these genera. Strains with the most pronounced secretion are a valuable resource for further biotechnological developments.

A comparison of bacterial diversity in samples analyzed using filter metabarcoding (205, 206) and in washouts from Petri dishes (w1–w3), as well as inoculation of bacteria during deposition from air (Sb) and from suspensions (Km) showed that the taxonomic composition for the latter is significantly different; however, in comparison with other variants, it is closest to the metabarcoding data obtained for filters. At the same time, despite the similar relative distribution of taxa, their number identified using standard 16S sequencing is, for obvious reasons, significantly inferior to metabarcoding, amounting to only about 10%.

## 5. Conclusions

The literature data and the data obtained in this work indicate the high importance of atmospheric microorganisms, which require large-scale multilateral research to understand the environmental processes occurring in the air, the possibility of using aerosol microorganisms for biotechnological or medical purposes, as well as to protect the population from possible infectious, allergic, and other diseases caused by them. The study of individual microorganisms is a necessary but not sufficient condition for understanding microbiological ecosystems. Metabarcoding sequencing and identification of microorganisms by culture methods are mutually complementary means of studying the atmospheric microbiome, allowing us to understand not only its taxonomic diversity, but also the functional potential and ecological role of the microorganisms that make up its composition. The use of metabarcoding sequencing of uncultured microorganisms in this work allowed us to significantly expand our understanding of the spectrum of the microbial population in the studied atmospheric aerosols.

The information obtained on the biodiversity and concentration of microorganisms in the atmospheric air of the Novosibirsk region is an important addition to the poorly studied characteristics of the biogenic component of the atmosphere of the urban environment of Novosibirsk and the suburbs.

## Figures and Tables

**Table 1:** Summary data on the diversity and abundance of microorganisms isolated from surface aerosols of atmospheric air by sorption method (Koltsovo settlement sampling).

Sample, Sampling Time	Medium of Isolation/Number of Microorganisms Isolated (CFU/mL)						
	LA	LA (1:10)	SAA	SA	Tg	Sabouraud Medium	Total Quantity
V1-1 8 September 2021 (day)	58 f 9 b, 12 n 4 akt, 6 k	44 f 38 b, 7 k, 28 n, 6 akt	25 f 2 b 6 akt 7 n	>1000 mch + akt	22 f 2 n 1 b	51 f 20 b	>1283
V2-1 8–9 September 2021 (night)	65 f 39 b, 13 k 7 n	58 f 60 b, 38 n	>100 fungi, bacteria count is impossible	>1000 mch + akt	12 f 5 n 3 d	50 f 30 b	>1450

Notations: f—fungi, b—spore-forming bacteria, n—non-spore-forming bacteria, k—cocci, akt—actinomycetes; mch—tiny colonies of unidentified, unculturable microorganisms.

**Table 2:** Summary data on the diversity and abundance of microorganisms isolated from surface aerosols of atmospheric air by filtration (Koltsovo settlement sampling).

Sample, Sampling Time	Medium of Isolation/Number of Microorganisms Isolated (CFU/mL)						
	LA	LA (1:10)	SAA	SA	Tg	Sabouraud Medium	Total Quantity
V1 8 September 2021 (day)	60 f 5 b 5 n	30 f 10 k	90 k 25 n 15 b	25 f	10 f 5 k	16 f	296
V2 8–9 September 2021 (night)	65 f 10 n	60 f 5 b	5 f	60 f	35 f 5 n	34 f 1 n	275

Notations: f—fungi, b—spore-forming bacteria, n—non-spore-forming bacteria; k—cocci.

## Data Availability

Data are contained within the article and Supplementary Materials.

## Supplementary Materials

The following supporting information can be downloaded at: https://www.mdpi.com/article/10.3390/microorganisms12102068/s1, Figure S1: Ratios of concentration (CFU/m^3^) of cultured microorganisms of different groups in samples of atmospheric aerosols of Novosibirsk and the region in 2021–2023 (% of the total number of isolates in the sample); Table S1: Climatic conditions during sampling. Meteorological parameters were recorded every minute using the Vantage Pro 2 meteorological complex (DAVIS, Italy), located at a height of approximately 4 m near the sampling point V, and all measured values were averaged over the sampling period. The average values ± mean square deviation are presented; Table S2: Results of the 16S taxonomic identification of cultured bacteria isolated from aerosol samples from Novosibirsk and the suburbs; Table S3: Relative abundance (%) of 16S rRNA gene amplicon sequences of different taxonomic levels for colony wash samples from agarized media with seeding of aerosol samples from 09/07–08/2021.
