# Supplementary material for: Three-Year Monitoring of Microorganisms’ Composition and Concentration in Atmospheric Aerosols of Novosibirsk City and Suburbs
Source: Microorganisms. 2024 Oct 15;12(10):2068. doi: 10.3390/microorganisms12102068 (PMC11509922; doi:10.3390/microorganisms12102068)
Supplement: Supplementary file 1 [file microorganisms-12-02068-s001.zip › microorganisms-3226628-supplementary.pdf]

Supplementary Materials

Figure S1. Ratio of concentration (CFU/mL) of cultured microorganisms of different groups in samples of atmospheric aerosols of Novosibirsk and the region in 2021-2023 (% of the total number of isolated in the sample).

Table S1. Climatic conditions during sampling. Meteorological parameters were recorded every minute using the Vantage Pro 2 meteorological complex (DAVIS, Italy), located at a height of approximately 4 m near the sampling point V, and all measured values were averaged over the sampling period. The average values ± mean square deviation are presented.

Table S2. Results of the 16S taxonomic identification of cultured bacteria isolated from aerosol samples from Novosibirsk and the suburb.

Table S3. Relative abundance (%) 16S rRNA gene amplicon sequences on different taxonomic levels for colony wash samples from agarized media with seeding of aerosol sample from 09/07-08/2021.

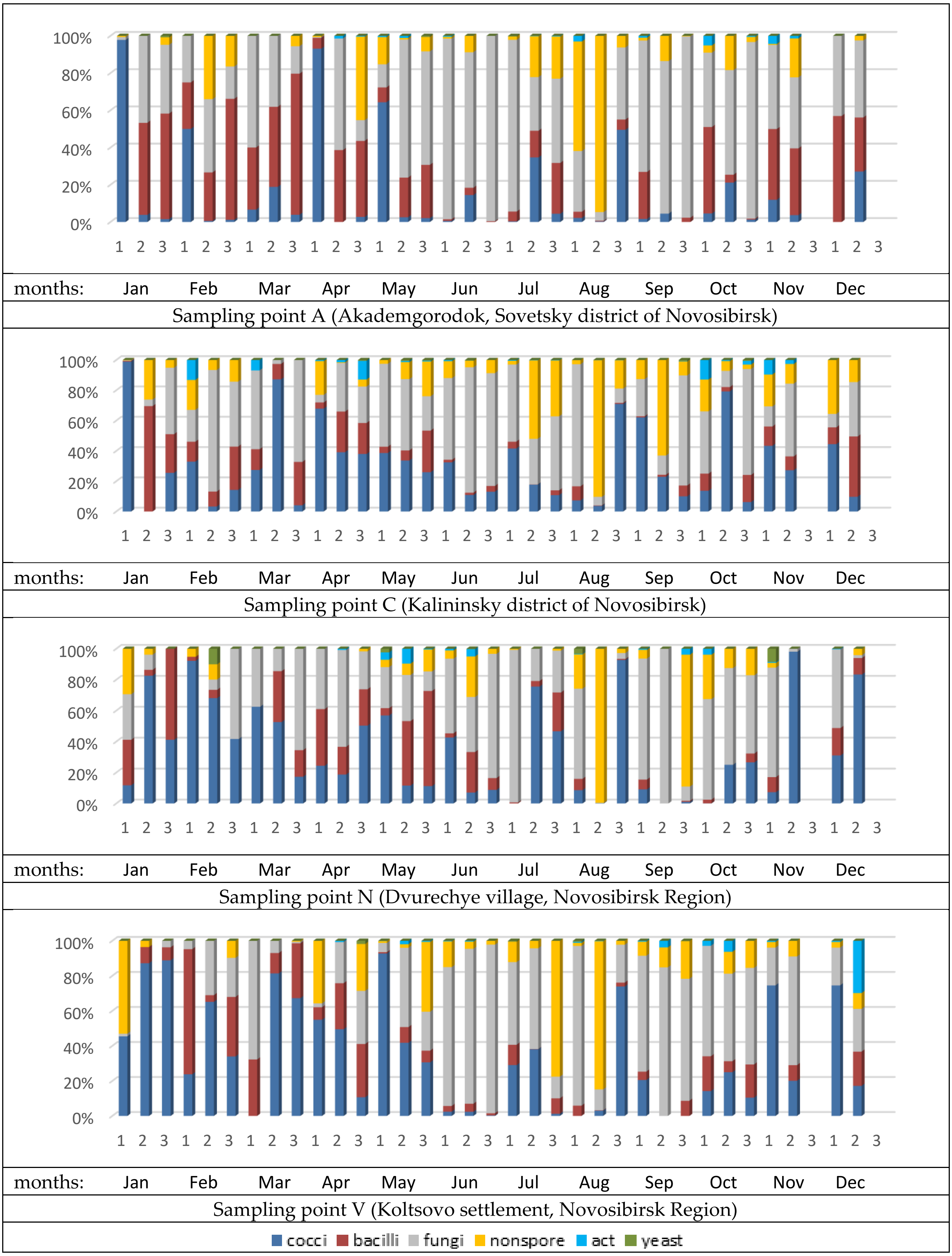

Figure S1. Ratio of concentration (CFU/m^3) of cultured microorganisms of different groups in samples of atmospheric aerosols of Novosibirsk and the region in 2021-2023 (% of the total number of isolated in the sample).

Denotations to **Figure S1**: 1 - ratio of microorganisms isolated in the samples of 2021; 2 - the same in the samples of 2022; 3 - the same in the samples of 2023.

Note: samples for November and December in 2023 are not available.

Table S1. Climatic conditions during sampling. Meteorological parameters were recorded every minute using the Vantage Pro 2 meteorological complex (DAVIS, Italy), located at a height of approximately 4 m near the sampling point V, and all measured values were averaged over the sampling period. The average values ± mean square deviation are presented.

Data	The time of sampling start 9 a.m.			Data	The time of sampling start 9 p.m.		
	Temperature, °C	Relative humidity, %	Wind velocity, m/s		Temperature, °C	Relative humidity, %	Wind velocity, m/s
23.09.2020	8.4 ± 0.8	77 ± 6	1.8 ± 1	23.09.2020	8.1 ± 0.3	78 ± 5	2.2 ± 1.1
07.10.2020	0.6 ± 0.5	90 ± 2	1.1 ± 0.7	07.10.2020	-1.0 ± 0.4	72 ± 3	0.7 ± 0.7
21.10.2020	12.1 ± 2.5	34 ± 8	2.1 ± 0.9	21.10.2020	10.2 ± 1.1	38 ± 4	3.0 ± 0.8
05.11.2020	1.7 ± 0.7	88 ± 2	2.8 ± 1.0	05.11.2020	1.7 ± 0.8	87 ± 3	1.3 ± 0.8
18.11.2020	-14.1 ± 0.8	79 ± 2	1.7 ± 0.7	18.11.2020	-13.9 ± 0.3	82 ± 1	1.2 ± 0.6
02.12.2020	-14.8 ± 1.3	83 ± 1	0.4 ± 0.5	02.12.2020	-13.3 ± 0.2	83 ± 1	0.0 ± 0.2
15.12.2020	-10.7 ± 1.5	86 ± 1	3.7 ± 1.2	15.12.2020	-13.4 ± 0.9	84 ± 0	1.4 ± 1.0
13.01.2021	-25.6 ± 1.8	73 ± 2	1.0 ± 0.9	13.01.2021	-27.4 ± 1.2	76 ± 1	0.5 ± 0.7
28.01.2021	-4.9 ± 1.9	89 ± 1	3.6 ± 0.9	28.01.2021	-6.1 ± 1.2	89 ± 0	2.5 ± 0.9
10.02.2021	-20.6 ± 0.7	69 ± 5	2.7 ± 1.4	10.02.2021	-25.9 ± 2.1	74 ± 2	1.9 ± 0.8
09.03.2021	-13.4 ± 0.7	65 ± 5	1.3 ± 1.4	09.03.2021	-21.5 ± 2.1	78 ± 2	0.0 ± 0.8
23.03.2021	0.1 ± 1.0	67 ± 7	1.2 ± 1.0	23.03.2021	-0.7 ± 0.9	79 ± 7	1.0 ± 0.8
06.04.2021	4.0 ± 0.5	73 ± 4	3.1 ± 1.1	06.04.2021	1.1 ± 1.2	75 ± 3	1.6 ± 0.9
20.04.2021	-2.9 ± 2.4	46 ± 5	1.9 ± 0.9	20.04.2021	-3.6 ± 0.4	58 ± 2	1.0 ± 0.8
04.05.2021	9.9 ± 1.6	46 ± 17	3.0 ± 1.4	04.05.2021	7.5 ± 0.9	46 ± 4	0.8 ± 0.8
18.05.2021	19.3 ± 2.5	68 ± 12	1.6 ± 1.2	18.05.2021	12.0 ± 1.6	92 ± 1	0.9 ± 0.8
01.06.2021	26.4 ± 2.0	32 ± 5	0.9 ± 0.8	01.06.2021	15.1 ± 3.3	71 ± 9	0.0 ± 0.1
15.06.2021	23.8 ± 2.2	51 ± 4	1.3 ± 0.9	15.06.2021	19.6 ± 1.7	70 ± 6	0.6 ± 0.6
29.06.2021	22.0 ± 2.5	55 ± 16	0.9 ± 0.8	29.06.2021	16.9 ± 1.4	80 ± 9	0.4 ± 0.6
13.07.2021	24.6 ± 2.0	64 ± 11	0.9 ± 0.8	13.07.2021	20.5 ± 1.3	79 ± 3	0.3 ± 0.5
27.07.2021	17.6 ± 1.6	61 ± 8	1.8 ± 1.1	27.07.2021	12.1 ± 2.5	84 ± 7	0.0 ± 0.0
10.08.2021	21.2 ± 1.8	77 ± 12	0.8 ± 0.9	10.08.2021	17.1 ± 1.3	86 ± 4	0.1 ± 0.2
24.08.2021	20.9 ± 2.9	70 ± 10	1.0 ± 0.8	24.08.2021	15.1 ± 1.9	90 ± 5	0.1 ± 0.3
07.09.2021	20.1 ± 2.3	58 ± 10	0.8 ± 0.8	07.09.2021	10.6 ± 2.0	88 ± 5	0.0 ± 0.1
21.09.2021	5.3 ± 0.8	83 ± 4	1.5 ± 0.9	21.09.2021	3.2 ± 0.7	88 ± 2	0.4 ± 0.6
05.10.2021	2.0 ± 1.1	92 ± 1	1.4 ± 0.8	05.10.2021	2.1 ± 1.6	82 ± 4	1.1 ± 0.8

19.10.2021	8.8 ± 3.0	57 ± 9	1.0 ± 1.1	19.10.2021	0.9 ± 3.0	79 ± 10	0.0 ± 0.0
02.11.2021	-12.6 ± 3.3	78 ± 2	2.0 ± 1.0	02.11.2021	-18.1 ± 0.2	82 ± 1	1.0 ± 0.8
16.11.2021	-10.5 ± 1.7	85 ± 1	0.1 ± 0.3	16.11.2021	-16.1 ± 1.8	85 ± 2	0.0 ± 0.0
30.11.2021	-6.8 ± 0.7	65 ± 2	1.6 ± 0.9	30.11.2021	-10.2 ± 1.1	77 ± 4	0.6 ± 0.6
14.12.2021	-16.7 ± 2.5	82 ± 2	0.6 ± 0.6	14.12.2021	-22.8 ± 3.0	79 ± 2	0.3 ± 0.6
28.12.2021	-10.2 ± 2.0	80 ± 2	1.9 ± 0.9	28.12.2021	-7.3 ± 0.3	81 ± 3	1.7 ± 0.9
11.01.2022	-15.2 ± 0.9	82 ± 2	1.6 ± 0.8	11.01.2022	-15.5 ± 0.9	82 ± 1	1.4 ± 0.8
25.01.2022	-16.3 ± 0.9	81 ± 1	0.9 ± 0.7	25.01.2022	-17.7 ± 0.4	80 ± 1	0.9 ± 0.7
08.02.2022	-13.0 ± 2.9	75 ± 4	1.0 ± 0.8	08.02.2022	-19.5 ± 1.9	82 ± 2	0.5 ± 0.7
22.02.2022	-2.9 ± 1.0	68 ± 3	1.4 ± 1.0	22.02.2022	-3.7 ± 0.5	73 ± 10	0.7 ± 0.6
09.03.2022	-13.3 ± 1.5	65 ± 5	1.3 ± 1.1	09.03.2022	-21.5 ± 1.8	78 ± 1	0.0 ± 0.0
22.03.2022	-2.9 ± 1.0	68 ± 3	1.4 ± 1.0	22.03.2022	-3.7 ± 0.5	73 ± 10	0.7 ± 0.6
05.04.2022	13.6 ± 2.0	44 ± 8	2.3 ± 1.0	05.04.2022	10.3 ± 2.2	59 ± 9	1.9 ± 1.3
19.04.2022	5.3 ± 2.0	37 ± 2	3.1 ± 1.4	19.04.2022	0.8 ± 1.9	61 ± 9	0.0 ± 0.1
03.05.2022	9.3 ± 1.0	42 ± 12	3.2 ± 1.1	03.05.2022	1.8 ± 3.2	55 ± 12	0.1 ± 0.3
17.05.2022	27.4 ± 2.2	32 ± 6	3.1 ± 1.1	17.05.2022	n.d.	n.d.	n.d.
31.05.2022	20.6 ± 2.0	54 ± 8	0.3 ± 0.5	31.05.2022	15.7 ± 2.0	81 ± 10	1.2 ± 1.0
14.06.2022	25.8 ± 2.9	54 ± 13	0.9 ± 0.9	14.06.2022	18.1 ± 1.3	80 ± 5	0.9 ± 0.9
28.06.2022	17.9 ± 0.9	66 ± 4	2.4 ± 1.4	28.06.2022	14.9 ± 1.6	79 ± 7	0.3 ± 0.5
12.07.2022	22.9 ± 1.4	54 ± 5	2.4 ± 1.1	12.07.2022	14.7 ± 2.5	77 ± 9	0.5 ± 0.7
26.07.2022	22.4 ± 2.5	63 ± 8	0.9 ± 0.8	26.07.2022	17.7 ± 1.8	88 ± 5	0.1 ± 0.2
09.08.2022	24.2 ± 2.9	57 ± 12	1.8 ± 0.8	09.08.2022	17.1 ± 2.4	81 ± 8	0.2 ± 0.3
23.08.2022	17.1 ± 1.8	57 ± 10	1.0 ± 0.9	23.08.2022	9.3 ± 2.0	90 ± 3	0.0 ± 0.0
06.09.2022	18.0 ± 3.0	63 ± 11	0.4 ± 0.6	06.09.2022	13.9 ± 1.3	87 ± 5	0.2 ± 0.3
20.09.2022	16.9 ± 2.4	55 ± 8	0.9 ± 0.9	20.09.2022	5.2 ± 2.4	89 ± 3	0.0 ± 0.0
04.10.2022	6.4 ± 3.4	56 ± 12	0.6 ± 0.6	04.10.2022	4.8 ± 0.9	64 ± 5	0.6 ± 0.7
18.10.2022	4.7 ± 0.6	88 ± 2	1.9 ± 1.0	18.10.2022	3.2 ± 0.2	90 ± 1	1.3 ± 0.8
01.11.2022	0.4 ± 1.5	84 ± 5	0.6 ± 0.6	01.11.2022	-0.6 ± 0.6	91 ± 2	0.2 ± 0.3
15.11.2022	3.0 ± 0.5	87 ± 2	2.3 ± 0.9	15.11.2022	0.9 ± 0.8	87 ± 4	4.3 ± 1.7
29.11.2022	-23.9 ± 2.5	72 ± 4	0.2 ± 0.3	29.11.2022	-28.3 ± 0.8	75 ± 0	0.0 ± 0.0
13.12.2022	-15.5 ± 2.9	82 ± 2	2.1 ± 1.0	13.12.2022	-24.6 ± 1.9	79 ± 1	0.0 ± 0.1
27.12.2022	-11.1 ± 1.3	72 ± 6	0.6 ± 0.8	27.12.2022	-11.4 ± 1.3	78 ± 6	0.3 ± 0.5
10.01.2023	-28.3 ± 0.4	67 ± 2	2.2 ± 1.1	10.01.2023	-29.8 ± 0.7	71 ± 0	2.1 ± 1.1

24.01.2023	-7.3 ± 0.9	90 ± 1	2.2 ± 0.7	24.01.2023	-7.6 ± 1.4	89 ± 1	1.1 ± 0.9
07.02.2023	-12.3 ± 1.7	79 ± 3	1.3 ± 1.0	07.02.2023	-18 ± 1.2	82 ± 2	0.7 ± 0.6
20.02.2023	-8.5 ± 2.5	79 ± 5	0.6 ± 0.8	20.02.2023	-11.7 ± 2.4	85 ± 3	0.1 ± 0.3
07.03.2023	-1.4 ± 0.9	70 ± 6	2.8 ± 1.8	07.03.2023	-0.5 ± 2.3	89 ± 7	2.0 ± 2.0
21.03.2023	-5.7 ± 1.6	54 ± 5	1.7 ± 1.3	21.03.2023	-13.2 ± 2.9	75 ± 6	0.0 ± 0.1
04.04.2023	6.7 ± 2.3	45 ± 3	2.2 ± 1.1	04.04.2023	-0.8 ± 2.5	75 ± 4	2.2 ± 1.0
18.04.2023	-7.2 ± 0.5	63 ± 15	3.3 ± 1.4	18.04.2023	-10.9 ± 1.1	56 ± 6	0.2 ± 0.3
02.05.2023	2.9 ± 2.1	65 ± 10	2.2 ± 1.0	02.05.2023	1.6 ± 1.5	73 ± 8	0.0 ± 0.1
16.05.2023	14.8 ± 2.0	34 ± 4	2.6 ± 1.0	16.05.2023	4.9 ± 3.5	79 ± 14	1.4 ± 1.6
30.05.2023	21.7 ± 2.7	35 ± 13	2.4 ± 1.3	30.05.2023	11.7 ± 4.8	63 ± 16	0.1 ± 0.2
12.06.2023	21.9 ± 2.4	46 ± 10	1.4 ± 1.0	12.06.2023	11.2 ± 2.9	87 ± 8	0.3 ± 0.7
27.06.2023	19.6 ± 2.2	61 ± 6	0.9 ± 1.1	27.06.2023	15.4 ± 1.3	87 ± 6	1.3 ± 1.2
11.07.2023	26.3 ± 2.9	65 ± 9	1.2 ± 0.9	11.07.2023	22.8 ± 0.9	84 ± 3	0.3 ± 0.5
25.07.2023	24.7 ± 1.3	61 ± 6	1.3 ± 1.0	25.07.2023	19.1 ± 2.1	78 ± 8	0.1 ± 0.4
08.08.2023	24.0 ± 1.7	56 ± 9	0.8 ± 0.9	08.08.2023	16.4 ± 2.7	85 ± 7	0.0 ± 0.1
22.08.2023	19.1 ± 2.9	72 ± 12	0.5 ± 0.6	22.08.2023	16.5 ± 0.9	81 ± 3	0.6 ± 0.8
05.09.2023	13.9 ± 0.6	83 ± 6	1.6 ± 1.0	05.09.2023	11.7 ± 1.1	75 ± 7	1.2 ± 0.8

Note: n.d. – no data are available.

Table S2. Results of the 16S taxonomic identification of cultured bacteria isolated from aerosol samples from Novosibirsk and the suburb.

№ п/п	Species name	Strain name
1.	*Arthrobacter bussei*	Kh-366
2.	*Arthrobacter cheniae*	Kh-379, Kh-380
3.	*Arthrobacter oryzae*	Kh-376
4.	*Agrococcus jenensis*	Kh-448
5.	*Acinetobacter schindleri*	Kh-454
6.	*Acinetobacter lwoffii*	Kh-457, Kh-455
7.	*Bacillus aerius*	Km-249, Km-330, Sb-84, Sb-69
8.	*Bacillus altitudinis*	Sb-74, Sb-69
9.	*Bacillus aryabhattai*	KB-434, Km-297, Km-303-2
10.	*Bacillus amyloliquefaciens*	Kh-489, Kh-490, Sb-03, Sb-94, Sb-95
11.	*Bacillus atrophaeus*	Km-341
12.	*Bacillus cereus*	Kh-453, Kh-492
13.	*Bacillus drentensis*	Km-367
14.	*Bacillus fengqiuensis*	KB-358
15.	*Bacillus firmus*	KB-347, KB-356, KB-338
16.	*Bacillus idriensis*	Km-263-1
17.	*Bacillus indicus*	Km-263-2
18.	*Bacillus licheniformis*	Kh-430, KB-454, KB-490, KB-491, KB-559, Sb-01, Sb-36, Sb-88
19.	*Bacillus massiliigabonensis*	Kh-456
20.	*Bacillus mobilis*	Kh-353, Kh-354, Kh-488, Kh-498, Kh-499, Kh-485, Sb-35
21.	*Bacillus mycoides*	Kh-412, Km-311, Km-312, Km-313
22.	*Bacillus paranthracis*	Km-343, Km-357
23.	*Bacillus proteolyticus*	Kh-487, KB-471, Km-315, Km-268
24.	*Bacillus pumilus*	Kh-349, Kh-350, Kh-429, Kh-491, Kh-419, Kh-421, Kh-425, KB-570, Km-303-1, Km-35, Sb-21a, Sb-02, Sb-16, Sb-62, Sb-91, Sb-93, Sb-22, Sb-81
25.	*Bacillus safensis*	Kh-363, KB-376, Km-254, Km-324, Sb-78
26.	*Bacillus simplex*	KB-545, Km-277
27.	*Bacillus stercoris*	Sb-21
28.	*Bacillus subterraneus*	KB-341, KB-353, KB-42
29.	*Bacillus subtilis*	KB-118, KB-550, KB-566, KB-568, Km-317, Km-322, Km-327, Km-339, Km-34, Sb-70, Sb-80, Sb-13, Sb-19, Sb-32
30.	*Bacillus tequilensis*	Km-346
31.	*Bacillus thioparans*	KB-340
32.	*Bacillus thuringiensis*	KB-470, KB-538, Km-276, Km-329, Km-338
33.	*Bacillus toyonensis*	Kh-357, Kh-497, Kh-494, Kh-486, KB-477
34.	*Bacillus tropicus*	Kh-496
35.	*Bacillus wiedmannii*	Kh-502, Kh-495, Km-328
36.	*Bacillus velezensis*	KB-54, Km-345, Km-348
37.	*Bacillus zhangzhouensis*	Km-285
38.	*Brachybacterium nesterenkovii*	KB-596
39.	*Brachybacterium ginsengisoli*	Kh-500
40.	*Brachybacterium alimentarium*	Kh-364, Kh-442, Kh-462
41.	*Brevibacillus reuszeri*	KB-72
42.	*Brevibacillus formosus*	KB-78
43.	*Brevundimonas nasdae*	Kh-371

44.	*Brevibacterium frigoritolerans*	KB-527
45.	*Glycomyces dulcitolivorans*	Kh-465
46.	*Corynebacterium ammoniagenes*	Kh-443
47.	*Corynebacterium mucifaciens*	KB-389, KB-377
48.	*Carnobacterium inhibens*	Kh-440
49.	*Curtobacterium flaccumfaciens*	Kh-394, KB-98, KB-598
50.	*Curtobacterium allii*	Sb-08
51.	*Curtobacterium plantarum*	Sb-71
52.	*Deinococcus proteolyticus*	KB-540
53.	*Exiguobacterium sibiricum*	Sb-23
54.	*Exiguobacterium acetylicum*	Sb-17, Sb-30, Sb-89
55.	*Exiguobacterium chiriqhucha*	Sb-39
56.	*Glutamicibacter ardleyensis*	Kh-424
57.	*Glycomyces dulcitolivorans*	Kh-464, Kh-466
58.	*Georgenia muralis*	Km-256
59.	*Frigoribacterium endophyticum*	Kh-426
60.	*Frigoribacterium endophyticum*	Kh-395
61.	*Frigoribacterium faeni*	Km-306
62.	*Kocuria rosea*	Kh-377, Kh-396, Kh-436, Kh-459, Kh-460, Kh-397, Kh-409, Km-251, Km-316
63.	*Lederbergia galactosidilytica*	Sb-14
64.	*Lysinibacillus fusiformis*	KB-68, KB-113, Km-257
65.	*Lysinibacillus manganicus*	KB-372
66.	*Massilia timonae*	Kh-369
67.	*Massilia oculi*	KB-555
68.	*Macrococcus caseolyticus*	Kh-479, Kh-431, KB-583
69.	*Microbacterium esteraromaticum*	KB-30
70.	*Microbacterium paraoxydans*	Kh-501
71.	*Microbacterium hydrocarbonoxydans*	Km-305, Km-308,
72.	*Mammaliicoccus lentus*	Kh-403, Kh-433, Kh-439, Kh-444, Sb-65
73.	*Mammaliicoccus vitulinus*	Kh-432, Kh-402
74.	*Microbacterium marinum*	Kh-372
75.	*Metabacillus idriensis*	Kh-360
76.	*Micrococcus endophyticus*	Km-272-1, Km-275
77.	*Neobacillus drentensis*	Kh-414
78.	*Niallia circulans*	Kh-418
79.	*Nocardiopsis quinghaiensis*	Kh-367, Kh-384, Kh-484, Kh-361, Kh-450, Kh-452, Kh-355, Kh-449
80.	*Paenarthrobacter nitroguajacolicus*	Kh-470
81.	*Paenibacillus lautus*	Kh-503, Kh-422, KB-163
82.	*Paenibacillus pabuli*	Kh-423
83.	*Paenibacillus provencensis*	KB-381
84.	*Paenibacillus glucanolyticus*	KB-457
85.	*Paenibacillus pabuli*	Km-318
86.	*Pantoea pleuroti*	Sb-05
87.	*Pantoea agglomerans*	Km-321, Km-326, Sb-75, Sb-92
88.	*Peribacillus butanolivorans*	Kh-358
89.	*Peribacillus frigoritolerans*	Kh-392, Kh-451, Kh-398, Kh-438, Kh-373, Kh-385
90.	*Planomicrobium okeanokoites*	Kh-359
91.	*Planococcus soli*	Kh-375
92.	*Planococcus massiliensis*	Sb-37
93.	*Planococcus versutus*	Kh-441

94.	*Planktothrix mougeotii*	Kh-415
95.	*Priestia aryabhattai*	Kh-381, Sb-10, Sb-48, Sb-04, Sb-26, Sb-31, Sb-41, Sb-79, Sb-87, Sb-15, Sb-20
96.	*Priestia aryabhattai/megaterium*	Kh-399, Kh-400, Kh-471, Kh-387
97.	*Pseudomonas baltica*	Kh-362
98.	*Pseudomonas mosselii*	Km-235, Km-242
99.	*Pseudomonas migulae*	Kh-468
100.	*Pseudomonas coleopterorum*	Kh-493
101.	*Pseudomonas putida*	Km-291
102.	*Pseudomonas helmanticensis*	Km-307
103.	*Psychrobacter maritimus*	Kh-404
104.	*Rothia endophytica*	Sb-66
105.	*Risungbinella massiliensis*	KB-382
106.	*Rhodococcus fascians*	Kh-445, Kh-446
107.	*Rathayibacter caricis*	Kh-428
108.	*Robertmurraya korlensis*	Sb-06
109.	*Rummeliibacillus stabekisii*	KB-441
110.	*Streptococcus thermophilus*	KB-588
111.	*Staphylococcus hominis*	KB-97, KB-546, Km-333, Km-337
112.	*Staphylococcus haemolyticus*	Kh-351, KB-552
113.	*Staphylococcus saprophyticus*	KB-528
114.	*Staphylococcus warneri*	Kh-352
115.	*Staphylococcus borealis*	Kh-378
116.	*Staphylococcus equorum*	Kh-405, Kh-434
117.	*Staphylococcus pseudoxylosus*	Kh-407, Kh-408, Kh-437
118.	*Staphylococcus pasteuri*	Kh-411
119.	*Solibacillus isronensis*	Sb-24
120.	*Sporosarcina aquimarina*	KB-549
121.	*Sporosarcina koreensis*	Kh-410
122.	*Stenotrophomonas tumulicola*	Km-236
123.	*Stenotrophomonas maltophilia*	Km-239
124.	*Streptomyces anulatus*	Kh-467
125.	*Stutzerimonas nitrititolerans*	Kh-504

Note: total data on selective genomic analysis of 270 bacteria isolated from the atmospheric air of Novosibirsk and the region of strains isolated over a three-year period; strain indices: KB, Km, Kh, Sb. 125 species, including 31 species of the genus *Bacillus* (112 strains), 37 strains of spore-forming bacteria of the genera *Lysinibacillus, Neobacillus, Paenibacillus, Peribacillus, Priestia, Sporosarcina, Solibacillus, Robertmurraya*.
Streptococci and *Staphylococci* - 16 strains of 9 species.
Kocuria rosea - 9 strains.

Table S3. Relative abundance (%) 16S rRNA gene amplicon sequences on different taxonomic levels for colony wash samples from agarized media with seeding of aerosol sample from 09/07-08/2021.

**Relative abundance (%) of phylums**							
	w.1	w.2	w.3		w.1	w.2	w.3
Firmiicutes	79	83	74	Bacteroides	0.9	0	3.1
Actinobacteria	14	1.7	20	Others	0.2	0	0
Proteobacteria	6.1	15	2.3				
**Relative abundance (%) of classes**							
	w.1	w.2	w.3		w.1	w.2	w.3
Bacilli	79	83	74	Chitinophagia	$9.2 \cdot 10^{-3}$	0	0.6
Actinobacteria	14	1.7	20	Sphingobacteriia	0.1	0	0.1
Gammaproteobacteria	2.4	13	0	Deinococci	0.2	0	0
Betaproteobacteria	3.2	2.1	2.0	Acidimicrobiia	0	0	0.1
Cytophagia	0.8	0	2.4	Flavobacteriia	0.1	0	0
Alphaproteobacteria	0.6	0.1	0.3	Chloroplast	0.0	0	0
				Clostridia	$2.0 \cdot 10^{-2}$	0	0
**Relative abundance (%) of orders**							
	w.1	w.2	w.3		w.1	w.2	w.3
Bacillales	79	83	74	Sphingobacteriales	0.1	0	0.1
Micrococcales	14	1.6	20	Deinococcales	0.2	0	0
Enterobacterales	1.1	13	0	Mycobacteriales	0.0	0.1	0
Burkholderiales	3.2	2.1	2.0	Acidimicrobiales	0	0	0.1
Cytophagales	0.8	0	2.4	Flavobacteriales	0.1	0	0
Pseudomonadales	1.2	0.4	0	unc_Chloroplast	0.0	0	0
Rhizobiales	0.3	0.1	0.3	Clostridiales	$2.0 \cdot 10^{-2}$	0	0
Chitinophagales	$9.2 \cdot 10^{-3}$	0	0.6	Propionibacteriales	$2.0 \cdot 10^{-2}$	0	0
Lactobacillales	0.2	0.1	0	Rhodospirillales	$2.0 \cdot 10^{-2}$	0	0
Rhodobacterales	0.3	0	0				
**Relative abundance (%) of families**							
	w.1	w.2	w.3		w.1	w.2	w.3
Bacillaceae1	56	77	60	Methylobacteriaceae	0.3	0	0.1
Micrococcaceae	12	1.6	20	Aerococcaceae	0.2	0.1	0
Bacillales	14	5.3	6.2	Rhizobiaceae	$2.0 \cdot 10^{-2}$	0.1	0.1
Erwiniaceae	1.1	13	0	Rhodobacteraceae	0.3	0	0
Planococcaceae	5.9	0.5	4.8	Bacillaceae2	0.0	0.0	0.1
Oxalobacteraceae	3.1	1.6	1.8	Sphingobacteriaceae	0.1	0	0.1
Paenibacillaceae1	1.5	0.7	2.3	Deinococcaceae	0.2	0	0
Hymenobacteraceae	0.8	0	2.4	Comamonadaceae	$9.2 \cdot 10^{-3}$	0	0.1
Staphylococcaceae	1.2	0	1.0	Iamiaceae	0	0	0.1
Microbacteriaceae	1.3	0	0.4	unc_Burkholderiales	0	0	0.1
Moraxellaceae	0.9	0	0	Burkholderiaceae	0.1	0	0
Pseudomonadaceae	0.3	0.4	0	Nocardiaceae	0	0.1	0
Chitinophagaceae	$9.2 \cdot 10^{-3}$	0	0.6	Flavobacteriaceae	0.1	0	0
Alcaligenaceae	0	0.5	0	unc_Clostridiales	$2.0 \cdot 10^{-2}$	0	0
Acetobacteraceae	$2.0 \cdot 10^{-2}$	0	0	Nocardioidaceae	$2.0 \cdot 10^{-2}$	0	0
**Relative abundance (%) of genera**							
	w.1	w.2	w.3		w.1	w.2	w.3
Bacillus	40	63	41	Pontibacter	0.0	0	0.4
Peribacillus	11	13	16	unc_Bacillaceae1	0.0	0.1	0.3
unc_Micrococcaceae	11	1.6	20	Microvirga	0.3	0	0.1
Exiguobacterium	14	5.3	6.2	Macrococcus	0.4	0	0
unc_Erwiniaceae	1.1	13	0	Domibacillus	0.4	0	0
Planococcus	4.5	0.4	3.2	Brevibacillus	0.1	0.0	0.2
Neobacillus	4.3	0	2.7	Aerococcus	0.2	0.1	0
Massilia	3.1	1.6	1.6	unc_Rhizobiaceae	$2.0 \cdot 10^{-2}$	0.1	0.1
Paenibacillus	1.4	0.7	2.1	Sphingobacterium	0	0	0.1

Hymenobacter	0.7	0	1.9	unc_Burkholderiales	0	0	0.1
Staphylococcus	0.8	0	1.0	Rhodococcus	0	0.1	0
Kocuria	1.6	0	0	Pedobacter	0.1	0	0
Solibacillus	0.1	0.1	1.2	Flavobacterium	0.1	0	0
unc_Microbacteriaceae	1.3	0	0	Paracnuella	$9.2 \cdot 10^{-3}$	0	0.1
unc_Planococcaceae	1.0	0	0	Caballeronia	0.1	0	0
Acinetobacter	0.9	0	0	Roseomonas	$2.0 \cdot 10^{-2}$	0	0
Psychrobacillus	0.3	0	0.4	Nocardioides	$2.0 \cdot 10^{-2}$	0	0
Pseudomonas	0.3	0.4	0	unc_Clostridiales	$2.0 \cdot 10^{-2}$	0	0
Achromobacter	0	0.5	0	Sphingobacterium	0	0	0.1
Microbacterium	0	0	0.4				
**Relative abundance (%) of OTUs**							
	w.1	w.2	w.3		w.1	w.2	w.3
OTU_2 (unc_Bacillus)	26	52	26	OTU_458 (unc_Rhizobiaceae)	$2.0 \cdot 10^{-2}$	0.1	0.1
OTU_78 (unc_Peribacillus)	11	13	16	OTU_95 (unc_Paracoccus)	0.3	0	0
OTU_14 (unc_Micrococcaceae)	11	1.6	20	OTU_866 (Paenibacillus_endophyticus)	0.1	0	0.1
OTU_15 (unc_Bacillus)	12	0	15	OTU_2562 (unc_Oxalobacteraceae)	0.1	0	0.2
OTU_135 (unc_Exiguobacterium)	11	4.6	2.7	OTU_432 (unc_Massilia)	0.1	0	0.1
OTU_50 (unc_Erwiniaceae)	1.1	13	0	OTU_4991 (unc_Chitinophagaceae)	0	0	0.2
OTU_7 (unc_Bacillus)	0	11	0	OTU_4337 (unc_Terribacillus)	0.0	0.0	0.1
OTU_68 (unc_Planococcus)	4.5	0.4	3.2	OTU_5312 (unc_Chitinophaga)	0	0	0.2
OTU_273 (unc_Exiguobacterium)	3.1	0.7	3.4	OTU_1098 (unc_Pontibacter)	0	0	0.1
OTU_289 (unc_Neobacillus)	4.3	0	2.7	OTU_903 (Cnuella_takakiae)	0	0	0.1
OTU_582 (unc_Massilia)	2.1	0	1.5	OTU_746 (unc_Psychrobacillus)	0.3	0	0.4
OTU_353 (unc_Paenibacillus)	1.0	0.4	1.3	OTU_1184 (Nibribacter_koreensis)	0.0	0	0.1
OTU_84 (unc_Massilia)	0.9	1.6	0	OTU_846 (Deinococcus_caeni)	0.1	0	0
OTU_179 (unc_Hymenobacter)	0.5	0	1.7	OTU_143 (unc_Pseudomonas)	0.1	0	0
OTU_766 (unc_Bacillus)	1.9	0	0	OTU_765 (unc_Bacillaceae1)	0	0.1	0
OTU_155 (unc_Staphylococcus)	0.8	0	1.0	OTU_446 (unc_Iamiaceae)	0	0	0.1
OTU_8 (unc_Kocuria)	1.6	0	0	OTU_2608 (unc_Sphingobacterium)	0	0	0.1
OTU_664 (unc_Solibacillus)	0.1	0.1	1.2	OTU_1128 (unc_Burkholderiales)	0	0	0.1
OTU_56 (unc Microbacteriaceae)	1.3	0	0	OTU_1836 (unc_Paenibacillus)	0	0	0.1
OTU_31 (unc_Acinetobacter)	0.9	0	0	OTU_407 (unc_Rhodococcus)	0	0.1	0
OTU_746 (unc_Psychrobacillus)	0.3	0	0.4	OTU_1352 (unc_Paenibacillus)	$2.0 \cdot 10^{-2}$	0	0.1
OTU_1716 (unc_Peribacillus)	0.5	0	0.2	OTU_99 (unc_Pedobacter)	0.1	0	0

OTU_2148 (unc_Paenibacillus)	0.1	0.3	0.2	OTU_662 (unc_Flavobacterium)	0.1	0	0
OTU_347 (unc_Pseudomonas)	0.2	0.4	0	OTU_311 (Paracnuella_aquatica)	$9.2 \cdot 10^{-3}$	0	0.1
OTU_2642 (unc_Achromobacter)	0	0.5	0	OTU_5583 (unc_Caballeronia)	0.1	0	0
OTU_93 (unc_Microbacterium)	0	0	0.4	OTU_735 (Deinococcus_ reticulitermitis)	0.0	0	0
OTU_584 (unc_Microvirga)	0.3	0	0.1	OTU_2777 (unc_Streptococcus)	0.0	0	0
OTU_194 (unc_Macrococcus)	0.4	0	0	OTU_724 (unc_Rufibacter)	0.0	0	0
OTU_397 (Hymenobacter_gelipurpu rascens)	0.2	0	0.1	OTU_133 (unc_Dietzia)	0.0	0	0
OTU_2220 (unc_Domibacillus)	0.4	0	0	OTU_4205 (unc_Pseudomonas)	0.0	0	0
OTU_1226 (unc_Brevibacillus)	0.1	0.0	0.2	OTU_1260 (unc_Streptophyta)	0.0	0	0
OTU_1882 (unc_Paenibacillus)	0.1	0.0	0.2	OTU_6780 (unc_Cupriavidus)	0.0	0	0
OTU_806 (unc_Bacillaceae1)	0.0	0	0.3	OTU_2173 (unc_Clostridiales)	$2.0 \cdot 10^{-2}$	0	0
OTU_161 (unc_Aerococcus)	0.2	0.1	0	OTU_282 (unc_Nocardioides)	$2.0 \cdot 10^{-2}$	0	0
OTU_1399 (unc_Pontibacter)	0.0	0	0.2	OTU_1731 (unc_Paenibacillus)	0.0	0	0.1
OTU_714 (unc_Rhizobacter)	$9.2 \cdot 10^{-3}$	0	0.1	OTU_4412 (unc_Fictibacillus)	0.1	0	0

Denotations: w.1, flush 1 (wach1, SA); w.2, flush 2 (wach2, LB); w.3, flush 3 (wach3, SA).
